# Strategies to enable workplaces to support autistic clinicians in the dental workforce: learnings from the Buckland Review of Autism Employment

**DOI:** 10.1038/s41415-025-9346-3

**Published:** 2026-03-27

**Authors:** Claire R. Newey, Jasmine F. Lewtas, Mary E. Voller

**Affiliations:** 252008120849725280638 Institute of Life Course and Medical Science, University of Liverpool, Liverpool, United Kingdom; 730182787213244929293 Formerly Institute of Life Course and Medical Science, University of Liverpool, Liverpool, United Kingdom

## Abstract

Embracing neurodiversity in the workplace can enhance productivity, job satisfaction, and ultimately create a more compassionate and effective healthcare environment. This can positively impact the wider dental team and overall patient care. Autistic individuals often encounter unique challenges in the workplace, particularly in fields like dentistry. This article focuses on the experiences of autistic dental clinicians, highlighting the barriers they face in employment and the potential need for reasonable adjustments in the workplace. Evidence such as the 2024 Buckland Review of Autism Employment emphasise that autistic adults often experience challenges securing and maintaining employment due to an absence of understanding and support. Legislations such as the Equality Act 2010, and other initiatives, including the Disability Confident Scheme and the Autistica Neurodiversity Employer Index, can protect autistic people in the workplace and help to overcome barriers. By implementing personalised adjustments specific to the individual needs of an autistic employee, such as sensory accommodations, flexible routines, and mentorship programmes, dental practices can cultivate a more inclusive environment. This article draws attention to the need for a dynamic shift towards valuing the unique strengths of autistic clinicians and integrating them into a diverse and equitable dental community.

## Key points

- To introduce the findings of the Buckland Review of Autism Employment to the dental workforce.
- To make readers aware of the challenges that autistic clinicians can encounter in the workplace.
- To make the dental team aware of the legislation that supports and protects autistic workers.
- To educate the wider dental team on strategies that can support autistic colleagues in the workplace.

## Introduction

Many autistic characteristics may be preferential in dentistry, such as attention to detail and hyperfocus. However, there is little research relating to the experience of autistic dentists, dental therapists and dental hygienists.

Awareness of autism is slowly improving in the school system and consequently more autistic people are being diagnosed in childhood. An estimated 1–2% of people in the UK are autistic; although, it should be noted that true figures are unknown.^1^ A large number of autistic adults, known as the ‘missed generation', remain undiagnosed, particularly those who do not relate to the autistic experience of the demographic upon which most of the research is based.^2^ Statistics also rely on data from formal diagnoses, and so do not include the portion of the autistic community who self-identify.^3^ Access to a diagnosis is a privilege which is not available to everyone.

For many autistic adults, the realisation that they are autistic arrives after seeking an autism assessment for their child. This, alongside the recent increase in awareness and understanding of autism and other neurotypes, is leading to more adults understanding that they are autistic, often after many years of struggles and misdiagnoses.

The Buckland Review of Autism Employment was published in February 2024.^1^ The purpose of the report was to identify the barriers autistic people have in securing ‘sustained and fulfilling employment' and to suggest ways to overcome those barriers. Only 30% of autistic people are in employment, compared to 50% of all disabled people and 80% of non-disabled people.^1^ In addition, only 35% of autistic employees fully disclose their diagnosis to their employer and 10% don't disclose to anyone at work.^1^

There are no data on how many dental practitioners are autistic; however, a recent study exploring the experience of medical doctors identified that 29% of respondents had not felt safe to disclose their autistic identity at work.^4^ Autistic people have a reduced life expectancy, and one of the major causes of death in autistic adults is suicide.^5^ In a study of autistic medical doctors, 77% of respondents had considered suicide, 24% had attempted it, and 49% had self-harmed.^4^ Autism is currently a highly politicised topic, both in the UK and internationally. The spread of misinformation by politicians and the media can contribute to the negative perceptions that autistic people face.^6^^,^^7^

The purpose of this article is to make dental practitioners aware of the findings of the Buckland Review and to provide suggestions of how the report's recommendations can be utilised in the dental workforce.

This article uses the terminology preferred by the autistic community: identity-first language. This refers to an autistic person, rather than a person with autism, although individuals may choose to use identify themselves using other descriptors.^8^ The article also refers to the roles of employers and employees. Many clinicians may be self-employed in their work setting; however, the legislation which protects disabled workers also covers self-employed workers such as dental associates.

## Autistic strengths and challenges

Autism is a developmental disability which affects how someone communicates with the world around them. Every autistic person will have their own unique combinations of strengths and challenges.

Autistic people may be acutely aware of their differences and how they are perceived socially or professionally; this can cause conscious or unconscious ‘masking' of these aspects of self within public settings which can lead to exhaustion and eventual burnout. Some autistic people may mask so effectively that their colleagues are not aware they are struggling in any way and may even appear to be thriving.

Many autistic people have pre-prepared mental scripts to help them navigate social and workplace settings. Autistic clinicians will often utilise these when communicating with patients and will be well-rehearsed at providing excellent patient interactions. However, they may struggle with social interactions outside of patient care if this hinges around small talk or unscripted interactions. This can pose an obstacle to fostering relationships with colleagues outside of clinical time and lead to isolation.

Some autistic people have meticulous attention to detail due to their above-average observational skills and have excellent recall. Hyperfocus or monotropism is a long-recognised and commonly stigmatised feature of autism, but within a dental setting it can allow a clinician to apply their skills with minimal distraction.

Autistic people are commonly stereotyped as lacking empathy; however, for many autistic people, empathy may be a particular strength. This is a characteristic that can be both a strength and a challenge for autistic clinicians, particularly those who are hyper-empaths. It can be an asset in a dental setting as it fosters development of strong professional relationships with patients; although, it can also be a source of great fatigue, which can follow the autistic clinician home.

Many autistic people have had to be highly resourceful in the workplace and may have finely tuned routines to help them to navigate and cope with the neurotypical world. This can mean that autistic people can be left without a key coping strategy if there is significant disruption to their routine or when they are in a new environment.

For some autistic clinicians, sensory issues and overstimulation triggers like bright lighting, or excess background noises may increase stress in the workplace. Autistic brains generate 41 times more information at rest than non-autistic brains, and autistic people are less able to ‘tune out' background sensory stimuli such as noise.^9^ This means that autistic people can become much more easily fatigued than non-autistic people from certain environments.

## Strategies to promote awareness, break down stigma, and enhance the contributions of autistic employees

In a National Autistic Society survey, 60% of employers reported worrying that they would get support for autistic people wrong and did not know where to seek advice about employing someone who is autistic.^1^

The Autistica Neurodiversity Employer Index is an initiative recommended in the Buckland Review.^1^^,^^10^ The Index promotes awareness via training materials, success stories, and best practices, and challenges misconceptions about autism in the workplace. It supports employers in making workplace adjustments to help autistic employees achieve their potential. Additionally, the Index highlights to employers how supporting the unique strengths of autistic employees can enhance productivity and contribute to a more dynamic, diverse workforce.

The Disability Confident Scheme is a government-led initiative that helps workplaces utilise the skills that disabled people can bring to the workforce. The scheme enables workplaces to receive recognition for adopting inclusive practices.^11^

## How employers can effectively support autistic clinicians beginning or returning to a career

Autistic clinicians may require additional support at points of transition, such as when leaving university and joining the workplace, when changing jobs, or when returning to a career. Employers can play a vital role in easing this transition period by providing personalised support mechanisms. Employment Autism is a charity that provides useful guides for employers, with information on recruiting, managing and developing autistic employees available on their website (employmentautism.org.uk).^12^

### Induction

Where possible, make information about the workplace available to the autistic staff member in advance. Sharing employment policies and procedures and general induction material will provide the autistic employee with information specific to this workplace, which can help them to prepare for their new job. The employee should have a job induction at the start of their new role and be welcomed to ask questions. Many autistic people will want to ask clarifying questions during this period to gain full understanding; employers should not view these questions as an attempt to question authority. Employees should have the opportunity to view their working environment if there was not opportunity during the interview process, for example, the specific surgery they will be working in.

### Mentorship

One of the most effective strategies to support an autistic colleague is to assign a mentor at the practice. This person should be able to offer appropriate support and has knowledge of the policies and procedures at the practice, such as a practice manager, partner or senior associate. Where possible, this mentor should have some autism training. It is crucial that the mentor assigned is someone that the autistic employee can trust and feel safe to have open discussions with. These discussions will help to create a tailored support plan which ensures that any adjustments put in place for the autistic employee are relevant and support their specific needs. Mentors should provide regular check-ins and should monitor the appropriateness of reasonable adjustments that are in place.

### Case study

‘*Transitioning from dental school to foundation training can be a challenging step, particularly for autistic dentists. I have found that reasonable adjustments have made a significant difference in supporting this transition. Simple things such as clear, step-by-step instructions, extra time to process information, access to quiet spaces to manage sensory overload, and the opportunity to ask questions without feeling rushed have all helped me feel more comfortable, build confidence, and focus on developing my clinical skills. These adjustments demonstrate how small, thoughtful changes can have a meaningful impact on supporting neurodivergent dentists in practice*' (autistic foundation dentist).

## Supporting autistic people already in the workforce

Adopting an inclusive and supportive environment for autistic clinicians is vital for improving their professional experiences and overall job satisfaction. A survey by the National Autistic Society found that half of autistic people surveyed identified acceptance, understanding, and support as the factors most crucial to help them thrive in the workplace.^1^

### Reasonable adjustments

Employers have a duty to provide reasonable adjustments under the Equality Act 2010.^13^ This Act also protects self-employed people such as dental associates. Reasonable adjustments are changes that can be implemented by employers to improve the experience and reduce disadvantages caused by a person's disability. As a hidden disability, there is potential for employers to overlook the support needs of autistic employees, and autistic people report that the burden is often on them to identify the adjustments they may benefit from.^14^ A personalised approach is essential, as no two autistic clinicians will have the same strengths or challenges. Instead of a one-size-fits-all strategy, creating tailored support plans is far more effective.

The Buckland Review revealed that poor execution of reasonable adjustments can present a significant barrier to work for autistic people.^1^ More than 25% of employees who asked for reasonable adjustments had their request refused, and more than one in ten reported adjustments were poorly implemented.^15^ Of a study of autistic doctors, only 46% of autistic doctors felt comfortable to request reasonable adjustments in their workplace, and the adjustments were implemented in only half of these cases.^4^ There is evidence to suggest that employers can perceive the act of requesting adjustments to be confrontational.^16^ Seeking adjustments often requires the autistic person to possess a strong autistic identity, as well as a disability identity, otherwise they can report feeling discouraged from requesting the adjustments that they need and are entitled to.^17^^,^^18^

Unsurprisingly, the failure of implementing adjustments can have significant impacts on the employee's wellbeing, and on the choices they make about their future career. This lack of support can lead to a sense of isolation, lower job satisfaction, and reduced productivity. These impacts can also affect mental health, leading to feelings of being overwhelmed or unsupported.^15^ The Buckland Review highlighted that recognising and supporting autistic people in the workplace is an important factor in increasing employee retention.^1^

Employers should be strongly encouraged to provide inclusive and adaptive working environments as good practice. Some examples of adjustments that could be easily implemented in a dental practice are shown in Table 1. Simple adjustments and flexibility may benefit all employees, not just those with additional needs. Many autistic clinicians may not feel comfortable disclosing their neurotype, but by employers adopting a flexible approach, employees can independently implement strategies which will help them to thrive in the workplace.^1^^,^^4^

**Table 1:** Examples of adjustments that could be implemented for workers in a dental practice setting

**Theme**	**Example of adjustment**
Sensory adjustments	Turning off the radio in the surgery Noise cancelling headphones or ear plugs to filter out background noise Screen adjustments, such as filters or anti-glare screens Lower-level ceiling lighting in clinic rooms Provide tools like ergonomic seating to improve comfort and focus Opportunity to choose own uniform or scrubs that are not, e.g., itchy or uncomfortable.
Fatigue	Regular breaks and offering these breaks at different times to colleagues if the autistic person want to be alone A quiet space to take breaks in.
Training	Colleague training to foster an understanding environment and reduce misconceptions.
Clear communication	Use written or visual guides for protocols, workflows, and procedural steps Structured feedback in a clear, constructive manner, avoiding ambiguous language Provide scripted explanations or visual aids to facilitate clear patient communication.
Routine and predictability	Maintaining a consistent schedule and giving advance notice of changes to allow for preparation and adaptation Working in the same surgery/ workspace to reduce changes and increase predictability.
Mental health support	(Via funding from Access to Work) Workplace counselling Peer support groups.

Autism is a dynamic disability, which means that an autistic person's support needs may vary at different times during their working career. Reasonable adjustments should be regularly reviewed, as individual adjustments may no longer be required or may need to be changed.

‘Buddy systems' can be used to cultivate a supportive work environment which fosters trusting relationships for employees to express clinical and personal needs. External bodies, such as NHS mentors and British Dental Association support, can provide valuable guidance on implementing this. Anonymous feedback mechanisms in the workplace can provide opportunity for staff to give feedback and request changes, which can help those who are hesitant to disclose their disability.

Creating a neuro-supportive environment in dental workplace settings can be further supported through the governmental ‘Access to Work' scheme. This scheme supports those starting or staying in employment by providing practical and financial assistance to those who require support and adaptations beyond what is considered reasonable by employers. This can include grants for additional support that exceeds the employer's duty.

### Case study

‘*My employer's reasonable adjustments have made a huge difference to my experience at work. Some have been more formal changes, like adding more structure to my day with planned rest breaks. During these breaks, I can use earplugs or noise-cancelling headphones to recharge after busy clinical sessions. I also have more flexibility around when I take annual leave, which helps me manage my energy levels and reduce the risk of autistic burnout. Other adjustments have been more informal, such as my colleagues developing a better understanding of autism, which has helped me feel accepted and supported. From an employer's point of view, I believe that making the right adjustments for staff with invisible disabilities like autism can really boost productivity and reduce sickness absence*' (autistic hospital dentist).

## How employers can adjust recruitment practices to meet the needs of autistic applicants

In accordance with the Equality Act 2010, employers also have a legal duty to make reasonable adjustments to the interview process to support autistic applicants.^13^ Providing pre-interview preparation materials and detailed information in advance about procedures and policies is important to autistic applicants. This may include details on the interviewers, the format, or duration of the interview, and can alleviate anxiety associated with uncertainty.

During the interview, using clear, unambiguous questions can help autistic candidates perform to their full potential. Sometimes, extra processing time may benefit autistic individuals to allow for extra time to formulate responses without rushing them. It can be helpful for autistic candidates if employers provide details of the next steps following the interview. This could include expected timescales for receiving feedback or the interview outcome.

## Positive implications for autistic dental patients

It is widely documented that autistic patients experience difficulties accessing healthcare and communicating with their healthcare providers.^19^^,^^20^ Informal reports in medicine suggest that communication between autistic doctors and autistic patients tends to be more effective than with allistic or neurotypical doctors.^21^ This is corroborated by research that indicates that autistic individuals often find it easier to interact with others who are also autistic, compared to non-autistic people.^22^^,^^23^ Autistic patients experience notable health inequalities compared with the general population, and autistic dentists and dental care professionals are uniquely positioned to offer valuable insights on potential ways to improve dental care and access for autistic people.^24^^,^^25^ Creating a workplace culture in dentistry where clinicians feel safe to disclose their autism without fear of discrimination has clear positive implications for autistic patients.

## Conclusion

Autistic people are often disadvantaged in the workplace and can face unique barriers to employment, with many struggling to access the reasonable adjustments they need to be able to thrive in the workplace without experiencing negative impacts on their health and wellbeing.

As a spectrum disorder, it is important to recognise that every autistic person has a unique combination of traits, experiences, strengths and challenges. Understanding this diversity and making workplace accommodations based on individual needs, can create a positive experience not only for the autistic employee but for the entire dental team. When adjustments are made it benefits everyone; employees work in a supportive environment, and patients receive care from a team that values and fosters diversity.

A workplace that is proactive in offering support to create an inclusive understanding environment is fundamental. By embracing neurodiversity, both employers and employees can cultivate a more collaborative and compassionate dental community, which enables professionals to thrive and supports mental health, efficiency, and job satisfaction.

Currently, within the UK dental workforce there is still work to be done to fully harness the valuable characteristics of autistic colleagues. The ongoing efforts to normalise and embrace neurodiversity will contribute to a more inclusive profession that values the unique strengths of all its members. By providing inexpensive and simple workplace adjustments in accordance with the Equality Act 2010, talented autistic team members will have the opportunity to flourish.^13^
